# The Impact of Water Sanitation and Hygiene (WASH) Improvements on Hand Hygiene at Two Liberian Hospitals during the Recovery Phase of an Ebola Epidemic

**DOI:** 10.3390/ijerph18073409

**Published:** 2021-03-25

**Authors:** Udhayashankar Kanagasabai, Kayla Enriquez, Richard Gelting, Paul Malpiedi, Celina Zayzay, James Kendor, Shirley Fahnbulleh, Catherine Cooper, Williamatta Gibson, Rose Brown, Nadoris Nador, Desmond E. Williams, David Chiriboga, Michelle Niescierenko

**Affiliations:** 1 John F. Kennedy Medical Centre, 1000 Monrovia, Liberia; 2 Emergency Medicine, School of Medicine, University of California, San Francisco, CA 94110, USA; Kayla.Enriquez@ucsf.edu; 3 Division of Global Health Protection, Centers for Disease Control and Prevention, Atlanta, GA 30329, USA; rug7@cdc.gov; 4 Division of Healthcare Quality Promotion, Centers for Disease Control and Prevention, Atlanta, GA 30329, USA; ffp4@cdc.gov; 5 Ministry of Health, 1000 Monrovia, Liberia; celinaozayzay@gmail.com (C.Z.); cthomascooper@gmail.com (C.C.); 6 Academic Consortium Combatting Ebola in Liberia, 1000 Monrovia, Liberia; jamesaakendor@yahoo.com (J.K.); shirna1@yahoo.com (S.F.); David.Chiriboga@umassmed.edu (D.C.); 7 Liberian Government Hospital, 2000 Tubmanburg, Liberia; williamattawilliamsgibson@gmail.com (W.G.); Luvcandace23@gmail.com (R.B.); 8 Centers for Disease Control and Prevention, 1000 Monrovia, Liberia; kzi6@cdc.gov (N.N.); zhx0@cdc.gov (D.E.W.); 9 University of Massachusetts Medical School, Worcester, MA 01655, USA; 10 Harvard Medical School, Boston, MA 02115, USA; Michelle.Niescierenko@childrens.harvard.edu; 11 Boston Children’s Hospital, Boston, MA 02115, USA

**Keywords:** water sanitation and hygiene (WASH), infection prevention and control (IPC), Ebola, low-resource setting

## Abstract

Fourteen years of civil war left Liberia with crumbling infrastructure and one of the weakest health systems in the world. The 2014–2015 Ebola virus disease (EVD) outbreak exposed the vulnerabilities of the Liberian health system. Findings from the EVD outbreak highlighted the lack of infection prevention and control (IPC) practices, exacerbated by a lack of essential services such as water, sanitation, and hygiene (WASH) in healthcare facilities. The objective of this intervention was to improve IPC practice through comprehensive WASH renovations conducted at two hospitals in Liberia, prioritized by the Ministry of Health (MOH). The completion of renovations was tracked along with the impact of improvements on hand hygiene (HH) practice audits of healthcare workers pre- and post-intervention. An occurrence of overall HH practice was defined as the healthcare worker practicing compliant HH before and after the care for a single patient encounter. Liberia Government Hospital Bomi (LGH Bomi) and St. Timothy Government Hospital (St. Timothy) achieved World Health Organization (WHO) minimum global standards for environmental health in healthcare facilities as well as Liberian national standards. Healthcare worker (HCW) overall hand hygiene compliance improved from 36% (2016) to 89% (2018) at LGH Bomi hospital and from 86% (2016) to 88% (2018) at St. Timothy hospital. Improved WASH services and IPC practices in resource-limited healthcare settings are possible if significant holistic WASH infrastructure investments are made in these settings.

## 1. Introduction

Water, sanitation, and hygiene (WASH) services are prerequisites for quality healthcare and are critically important for the safe management of infection prevention and control (IPC) [1]. One in four health facilities around the world lack basic water services, and one in five have no sanitation services, impacting approximately 2 billion people worldwide [1]. In 2016, 45% of healthcare facilities in the least-developed countries had no basic water, 21% had no sanitation service, and 27% had basic waste management services [2]. WASH infrastructure varies considerably between regions, with more than one in four healthcare facilities in sub-Saharan Africa lacking access to water [1]. The WHO–UNICEF Joint Monitoring Programme for WASH estimated that 896 million people use healthcare facilities with no water services and 1.5 billion use facilities with no sanitation services [2]. This paucity of WASH infrastructure and services compromises the delivery of basic routine health services, such as obstetric deliveries and immunizations, as well as the ability to prevent and control infections. More than 1 million deaths each year are associated with unclean births, while infections account for 26% of neonatal deaths and 11% of maternal mortality in resource-limited settings [1]. Unsafe management of healthcare waste presents other health risks as well, exposing healthcare workers (HCWs), waste-handlers, patients, their families, and communities to preventable infections, toxic effects, and injuries [3]. A lack of IPC can also contribute to antimicrobial resistance (AMR), a growing health threat worldwide.

The main objective of WASH programs in disasters is to reduce exposure to disease-bearing pathogens or vectors, thus reducing the transmission of disease [4]. The Ebola virus disease (EVD) outbreak in West Africa that began in 2014 was the largest and most devastating since the Ebola virus was first discovered in 1976 in the Democratic Republic of the Congo [5]. The epidemic had a global impact; however, the hardest hit countries were Guinea, Liberia, and Sierra Leone in West Africa [6,7].

### 1.1. Assessing the Need

Liberia’s 2014 EVD outbreak occurred as the country was still recovering from a 14-year civil conflict that devastated infrastructure countrywide and left the healthcare system shattered [8]. Healthcare facilities were ill-designed, poorly equipped, and not prepared to provide the necessary occupational and patient-safety practices required for the delivery of safe and effective health services. The outbreak placed an incredible strain on a system which lacked public-health infrastructure, including appropriate IPC measures, critical WASH infrastructure and supplies, accessible healthcare facilities, and well-trained infection control professionals [5,9]. The shortage of personal protective equipment (PPE) and IPC supplies, poor WASH infrastructure and services, and a lack of compliance with basic IPC measures contributed to 372 healthcare workers acquiring the Ebola virus disease in Liberia, of whom 184 died [10]. EVD not only exposed the weaknesses of the health system but also revealed the public’s lack of confidence and trust in the safety of the health system. In the early stages of the outbreak, multiple health facilities shut down because of poor IPC practice, knowledge of HCWs, and infrastructure, throwing a spotlight on the limitations of the Liberian health system. Between August and December 2014, outpatient visits were 61% lower and antenatal care visits 40% lower than the same timeframe in 2013 [10].

In September 2014, the Liberian Ebola response national IPC Task Force was established, chaired by the Ministry of Health (MOH) and supported by international partners and local nongovernmental organizations (NGOs). The Task Force rapidly developed national policies and guidelines for IPC practices in general healthcare facilities [9]. The pre-existing structural vulnerabilities of the health system and limited health workforce capabilities hindered an effective response to the epidemic and contributed to the scale of the outbreak [11]. Post-EVD outbreak, the MOH identified high-quality health-service delivery with re-engineered health infrastructure as one of nine key investment areas [11]. The United Nations Children’s Fund (UNICEF), in collaboration with the MOH, conducted a gap analysis in the last quarter of 2015, which identified and prioritized major WASH improvements at 45 public-health facilities, including health centers and hospitals. The main government hospitals in 10 out of 15 of Liberia’s counties were among the highest priority facilities identified for WASH interventions.

**Figure 1:**
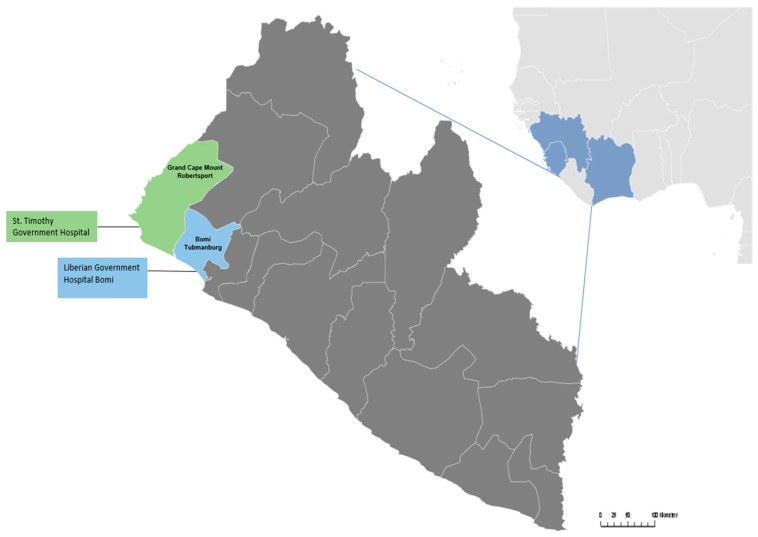
Location of St. Timothy Hospital and Liberian Government Hospital Bomi.

The Academic Consortium Combating Ebola in Liberia (ACCEL), an academic consortium made up of physicians from U.S. universities and the Liberia College of Physicians and Surgeons (LCPS), was among the NGO partners tasked with providing necessary IPC supplies and WASH support to hospitals. ACCEL took a multipronged approach to its response work, developing a mentorship and quality-improvement program that reinforced previous IPC training, along with integrating aspects of supply chain improvement and WASH into programming. In consultation with the MOH and UNICEF, ACCEL also agreed to undertake comprehensive WASH renovations in Liberia Government Hospital Bomi (LGH Bomi) and St. Timothy, two of the county hospitals (Figure 1).

Liberian Government Hospital-Bomi (LGH Bomi) is in the city of Tubmanburg in Bomi County. Bomi County is located in the western region of the country. During the EVD outbreak, Bomi County saw the sixth highest number of EVD cases in the country, 332 (suspected, probable, and confirmed), and 177 cumulative deaths caused by EVD. The hospital was continually open and provided services during the outbreak, despite lacking pipe-borne water and proper waste-disposal mechanisms and having poor IPC practices.

St. Timothy Government Hospital (St. Timothy) was the first hospital built in Liberia, in 1917, by the Episcopal Church, and is located on a steep hill in the coastal city of Robertsport, Grand Cape Mount County, in the western part of Liberia. It shares a border with Sierra Leone. During the EVD outbreak, the county saw a total of 445 cases (suspected, probable, and confirmed) and 315 cumulative deaths, making it the county with the fifth highest number of cases. Despite having to provide care to high-risk patients, the hospital functioned without pipe-borne water and proper waste-disposal mechanisms and with limited IPC practices.

Results from the UNICEF–MOH gap analysis for these two hospitals is shown in Table 1. The U.S. Centers for Disease Control and Prevention (CDC), through Global Health Security Agenda (GHSA) programming, also helped to provide technical assistance and funding for these ACCEL efforts.

### 1.2. Guidance and Standards

WASH in healthcare facilities is a fundamental prerequisite for achieving national health goals and sustainable development goals (SDGs) to ensure healthy lives and promote well-being (SDG 3) and to ensure the availability and sustainable management of water and sanitation (SDG 6). Access to safe water; the presence of functioning handwashing facilities and latrines; hygiene and cleaning practices; and appropriate sorting and disposal of waste are especially important for improving health outcomes linked to maternal, new-born, and child health and for carrying out basic IPC practices necessary to prevent healthcare-associated infections (HAIs). In order to help ensure the presence of these factors, in 2016, the MOH, in collaboration with other ministries, developed the “WASH and Environmental Health Package in Health Facilities” [12]. The package included hardware and software components. The hardware portion aimed at improving the quality and quantity of WASH facilities in healthcare settings via the rehabilitation of water points, toilets and wastewater collection systems, handwashing facilities, and laundry and mortuary facilities. The software portion focused on improving and managing the WASH facilities to avoid nosocomial infections among HCWs, patients, and the community though hand hygiene practices, behavioural change, and communication. This guidance document includes minimum recommended environmental health and WASH requirements for healthcare facilities in Liberia and is aligned with global standards. It was developed as part of Liberia’s program for early recovery and building a resilient healthcare system for transitioning from EVD response to improving the quality of care within routine health services. The package includes the use of WASH Safety Plans, which align with WHO’s WASH Facility Improvement Tool (WASH FIT), a risk-based, continuous improvement framework with a set of tools for undertaking WASH improvements as part of wider quality improvements in healthcare facilities [13]. As part of the comprehensive WASH renovations undertaken by ACCEL and funded by the U.S. CDC, the WASH FIT process was also implemented at Liberia Government Hospital Bomi and St. Timothy Government Hospital. WASH renovations undertaken at the two hospitals also complied with guidelines from the MOH Infrastructure Unit, including the use of standard designs for WASH components for healthcare facilities.

## 2. Materials and Methods

In this paper, we present the results of a pre/post-interventional study conducted at two rural hospitals in Liberia from September 2015 to September 2018. Comprehensive WASH renovations, as well as mentoring and supply chain improvements to reinforce IPC practices, were implemented in these two hospitals during this timeframe. To measure the impact and outcomes of the intervention, we collected pre/post data on minimum Liberian WASH standards, hand hygiene (HH) compliance, and the cost of implementing the intervention.

### 2.1. WASH Minimum Standards

Baseline WASH facility observations were conducted at Liberia Government Hospital Bomi and St. Timothy Government Hospital from November 2015 to January 2016 by trained Liberian WASH technicians that represented both the ACCEL and MOH. Information collected was based on minimum standards set forth by the Liberian WASH and Environmental Health Package in Health Facilities and targets set by SDG 6 [2,12].

### 2.2. Hand Hygiene Audits

Pre- and post-intervention observation of healthcare-worker hand hygiene compliance was conducted at three time-points before (October 2016), during (June 2017), and after (March 2018) intervention over a week, both during day shifts and night shifts, as part of the MOH’s routine hand hygiene auditing program. All observations were conducted at random for two to three days by external Liberian HCWs, without the facility HCWs’ knowledge that they were being monitored. The three enumerators performing the audits had received extensive training in data collection and IPC best practices. Observations of healthcare workers occurred in their routine clinical environment during both day and night shifts. The standard WHO hand hygiene audit tool [14], which had been disseminated in Liberia for use in healthcare facilities, was used to collect the data.

### 2.3. Cost Analysis

Cost information on renovations conducted at the hospitals was collected for each of the two sites. Data collected included the expenditures on renovation materials, equipment, long-term service contracts for the equipment, staff oversight costs, and the cost of material transportation to the sites. Beyond implementation costs, data were collected on the time frames to implement interventions, as well the type of WASH renovations conducted.

### 2.4. Data Analysis

WASH minimum standard assessments were compared pre- and post-intervention to evaluate for compliance with guidelines, and overall changes were documented. HH observation results were entered into Microsoft Excel and were analyzed using descriptive statistics to evaluate HH compliance and assess the trend in compliance over the course of the intervention. Three different compliance measures were evaluated: (1) overall HH compliance, defined as proper technique and use of alcohol-based hand rub or soap-and-water handwashing performed by HCWs, before and after a single patient-care occurrence; (2) trends in both pre- and post-patient-care handwashing as a marker of healthcare workers’ motivations to protect themselves versus patients; and (3) HCW utilization of alcohol-based hand rub versus that of soap-and-water handwashing as a preferred method of HH.

### 2.5. Ethical Considerations

The hospital HH audits and WASH FIT assessments were part of the MOH standard clinical programs and were exempt from IRB review. As a partner NGO, permission was obtained from the MOH and facility administrators to conduct the assessments and audits. Healthcare workers were aware this was part of government health facility standard operating procedure. No individual HCW or patient data were collected at any time.

## 3. Results

### 3.1. WASH Minimum Standards

Results from the initial UNICEF–MOH gap analysis showed that neither LGH Bomi nor St. Timothy met minimum WASH requirements (Table 1). To address these needs, a total of 28 infrastructure-related improvements were identified at each hospital, and comprehensive WASH renovations were successfully completed at both facilities (Table 2). In the domains of water, sanitation, hygiene, and waste management, services were improved from no service to basic services at both intervention sites (Table 3).

### 3.2. Hand Hygiene Audits

**Figure 2:**
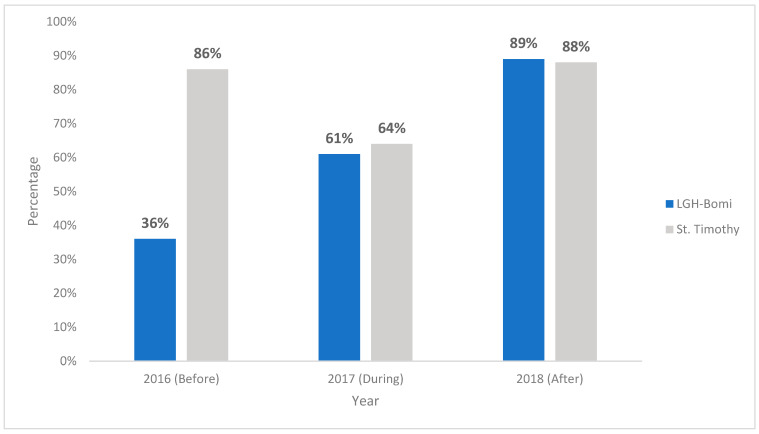
Overall hand hygiene* compliance before, during, and after intervention. * Overall hand hygiene is the proper technique and use of alcohol-based hand rub or soap-and-water handwashing before and after a single patient-care occurrence.

**Figure 3:**
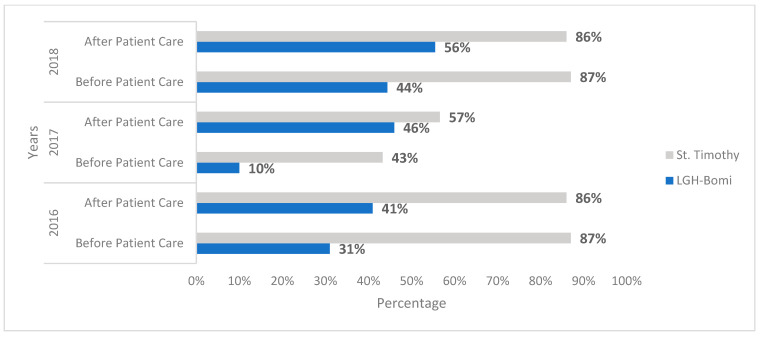
Hand hygiene compliance before and after patient care at two hospitals in 2016, 2017, and 2018—before, during, and after intervention.

**Figure 4:**
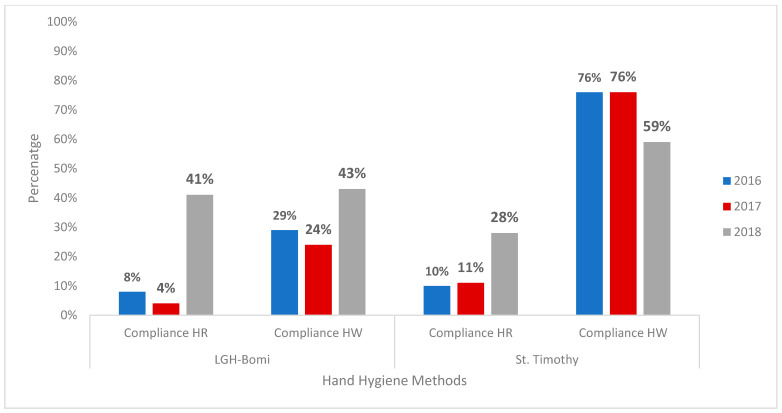
Alcohol-based hand rub and handwashing compliance at LGH Bomi and St. Timothy, 2016 to 2018. Abbreviations: HR = alcohol-based hand rub, HW = handwashing.

Hand hygiene audits conducted at LGH Bomi and St. Timothy in 2016 before the WASH-improvement interventions showed a baseline overall HH compliance of 36% and 86%, respectively. During the renovation period, an increase to 61% HH compliance was noted at LGH Bomi and a drop from the high baseline to 64% was noted at St. Timothy. In 2018, after WASH improvements, HH audits conducted showed an overall HH compliance of 89% and 88% at LGH Bomi and St. Timothy, respectively (Figure 2). Healthcare-worker practice of HH before and after patient contact was observed at the same three time points: 2016 (before the intervention), 2017 (during the intervention), and 2018 (after the intervention). HH compliance before patient contact in 2016 was 31% at LGH Bomi and 87% at St. Timothy; in 2017, it was 10% at LGH Bomi and 43% at St. Timothy; in 2018, it was 44% at LGH Bomi and 87% at St. Timothy (Figure 3). HH compliance after patient contact in 2016 was 41% at LGH Bomi and 86% at St. Timothy; in 2017, it was 46% at LGH Bomi and 57% at St. Timothy; in 2018, it was 56% at LGH Bomi and 86% at St. Timothy (Figure 3). In both health facilities, hand rub compliance was lower (8%, 4%, and 41% in LGH Bomi and 10%, 11%, and 28% in St. Timothy before, during, and after intervention, respectively) compared to handwashing compliance (29%, 24%, and 43% in LGH Bomi and 76%, 76%, and 59% in St. Timothy before, during, and after intervention, respectively) (Figure 4).

### 3.3. Implementation Cost

The costs of renovations (renovation materials, equipment, solar installations, long-term service contracts for the equipment) (Table 2) and the overall cost of implementation (including staff oversight costs and transportation to the site) are summarized in (Table 4). The total costs of the renovations were USD 348,796.74 at LGH Bomi and USD 382,027.96 at St. Timothy. The costs of the renovation contracts were USD 174,155.78 (50.0% of total costs) and USD 194,057.00 (50.8% of total costs); the costs of equipment purchased for each facility were USD 55,259.00 (15.8% of total costs) and USD 67,989.00 (17.8% of total costs), and the costs of service contracts to maintain the equipment for five years were USD 8400 (2.4% of total costs) and USD 9000 (2.4% of total costs) at LGH Bomi and St. Timothy, respectively. The costs of oversight included the salaries of WASH technicians and engineers who assessed the sites, coordinated the WASH FIT process, and traveled to the two hospitals located two to four hours from the capital city. The total costs of training each facility’s healthcare workers in the MOH-endorsed WASH curriculum were USD 10,200 at LGH Bomi and USD 10,200 at St. Timothy. The total costs of mentoring each facility’s healthcare workers every month for nine months after the training were USD 6750 at both LGH Bomi and St. Timothy. The timeline for implementation of the project was two years in total. Project approval was secured in September 2016 from the CDC; renovations started in March 2017, and the project was handed over to the government of Liberia in September 2018.

## 4. Discussion

This study describes comprehensive WASH renovations conducted at two rural hospitals during the recovery phase of the world’s largest EVD outbreak in Liberia and the intersection of those interventions with hand hygiene behavior. Baseline assessments showed that WASH infrastructure was inadequate compared to Liberian standards at the two facilities assessed, although these standards are unmet at the majority of public health facilities in the country. Overall, the interventions improved existing WASH infrastructure to standards as recommended by WHO’s “WASH in Health Care Facilities” [1], as well as to Liberian standards [12].

A key finding of this study is that it demonstrated how combined infrastructure improvements and IPC training and mentoring can lead to improved IPC practices. Specifically, it showed that healthcare-worker IPC practices like HH that require infrastructure improved when infrastructure investments were made alongside behavioral-change efforts. This was demonstrated by improvements in overall hand hygiene at both facilities, as well as pre-patient-care and post-patient-care hand hygiene at LGH Bomi, over the course of implementation of comprehensive WASH renovations at the two facilities.

LGH Bomi was able to demonstrate a larger margin of improvement in hand hygiene compliance (36% to 89%), whereas St. Timothy was able to achieve a smaller margin of improvement (86% to 88%). The large margin of improvement seen at LGH Bomi could be attributed to several factors such as the ease of accessibility, leadership, more intense supervision from the county health team IPC focal person, and the location of the hospital (in close proximity to the county health office). Despite an overall improvement, the lack of more significant improvement in St. Timothy hospital could be partially explained by attrition in staffing, variability in staff observed, and availability of supplies in this remote location, which would have hindered improvement. The increased HH compliance after patient care versus before patient care highlights HCW’s perceptions of personal safety after patient interactions as being of importance. This protective behavior of HCWs could be a result of fear of contracting EVD, following the high number of HCWs who contracted EVD during the epidemic. This finding should be taken into consideration when designing future training programs for HCWs in relation to patient safety.

This study has several implications. First, WASH infrastructure improvements during recovery from an emergency response are possible and feasible given suitable circumstances, as discussed further below. Second, this work highlights the need for strengthening national policies, guidelines, and standards so that there are established targets to meet. Third, sufficient financing, especially for large infrastructure improvements, though costly, is necessary; however, strategic use of donor financing can help to overcome this investment hurdle. For example, in this case, linking the work to the larger initiative of global health security helped ensure adequate funding for WASH renovations during the recovery phase. Finally, the data were analyzed using descriptive methods using Microsoft Excel, and further complex analysis was not done because of the quality of data collected.

We found several considerations related to sustainability of the intervention and change in practice. The need for sufficient financing extended to other factors such as workforce capacity and the sustainability of IPC practices. In this case, trained technical staff was needed onsite at these remote locations to ensure the successful completion of WASH renovations by contractors. This investment was key to ensuring high-quality materials and craftsmanship and will improve the durability of the infrastructure improvements. The use of solar-powered water pumps, though costly as an investment, will require lower operating costs in the long term. In addition, the inclusion of service contracts for new equipment will help to extend the life of the equipment through proper maintenance.

### Limitation

This study has several limitations caused by the type of data that could be collected in the Liberian setting. For example, at the time of the implementation of the intervention, there was limited capacity in Liberia to document culture or PCR-confirmed HAIs, so HAI rates are unknown. Therefore, observed changes in HH practices are assumed to positively impact the number of HAIs, on the basis of prior research [15]. These changes in HH practice cannot be attributed solely to infrastructure improvements, as ongoing countrywide IPC training programs by various partners and the MOH prior to the intervention may have also contributed to improved HH compliance. Other factors also likely contributed to HH compliance and improvement, such as the improved availability of soap and alcohol-based hand rubs from NGOs and partner donations, and the introduction of new protocols and procedures by the WHO and MOH. Response bias, especially the alteration of behavior due to being observed (i.e., the Hawthorne effect) may have also influenced HH results. This effect may be particularly relevant for St. Timothy Government Hospital, where baseline hand hygiene compliance was 86%, 20 percentage points above the national average for government hospitals.

## 5. Conclusions

This study describes one of the few documented combined WASH and IPC interventions that integrated WASH renovations alongside IPC training and hospital mentorship. Prior research has typically only documented how inadequate environmental conditions in healthcare facilities (e.g., poor WASH, lack of ventilation, inadequate management of healthcare waste) can result in HAIs [15,16,17]. Additionally, studies have also documented the impact of HH and other IPC practices on HAIs [15,18,19]. This complex intervention combined these approaches, assessing not only changes in HH but also compliance with international environmental health standards for healthcare facilities and the cost and investment value in this approach.

It is critically important to consider infrastructure investments in WASH for healthcare facilities in resource-limited settings in order to improve IPC practices and to avoid the amplification of epidemics in health facilities. In the fields of IPC and WASH, education and training alone may be insufficient to improve practice. Instead, to make a meaningful and sustainable impact, we must consider other factors. Those factors include strengthening national policies and standards and ensuring sufficient financing to improve and sustain facilities and maintain the presence of trained staff and staff mentoring. Further WASH and IPC investments by the Liberian government and local stakeholders are essential to sustain and improve the quality of care provided at health facilities. The incorporation of IPC and WASH training programs into the curricula of institutions of higher learning is one way to ensure that future generations of HCWs are knowledgeable about best practices within the health system. WASH standards that meet WHO minimum global standards and/or local standards for healthcare facilities are vital to improving IPC practices and reducing hospital-acquired infections in healthcare facilities. WASH investments in the health sector should remain a priority for governments and international donor agencies, despite the barriers in undertaking such projects.

## Figures and Tables

**Table 1:** Gap analysis of Liberian Government Hospital Bomi and St. Timothy—Liberia, 2016.

**Liberian Government Hospital Bomi**					
Water Supply		Sanitation		Waste Disposal	
***Element Assessed***	***Findings***	***Element Assessed***	***Findings***	***Element Assessed***	***Findings***
Water Source	Yes	Toilet type	No	Incinerator	Poor
Water Treatment	No	Septic tank	No	Placenta pit	No
Water Storage	Inadequate	Drainage	No	Ash pit	Required rehabilitation
Water Distribution	Faulty	Bathrooms	No	Sharps pit	No
Water Points	No	Soak Pits	No		
Internal Plumbing	No				
**St. Timothy Hospital**					
Water Supply		Sanitation		Waste Disposal	
***Element Assessed***	***Findings***	***Element Assessed***	***Findings***	***Element Assessed***	***Findings***
Water Source	Unprotected	Toilet type	Indoor flush	Incinerator	Poor
Water Treatment	Not Indicated	Septic tank	Not Indicated	Placenta Pit	Not Functional
Water Storage	Not Functional	Drainage works	Not Indicated	Ash pit	Not Functional
Water Distribution	Poor	Bathrooms	Not Indicated	Sharps pit	None
Water Points	No	Soak Pits	Not Indicated		
Internal Plumbing	No				

**Table 2:** Water, sanitation, and hygiene (WASH) domain, intervention, and estimated and actual costs at Liberian Government Hospital Bomi and St. Timothy Government Hospital—Liberia, 2016.

Domain	Interventions *	Estimated Cost (USD)	Implementation Cost (USD)
Liberian Government Hospital Bomi			
Water	- Concrete water tower (capacity 12,000 gallons) - Drilling of new borehole - Water transmission and distribution systems including fixtures - Generator (80 kilovolts) - Solar-powered submersible pumps	$172,528.76	$194,057.00
Sanitation	- Rehabilitate in-patient toilets - Rehabilitate leaking sewer lines - Rehabilitate the storm drainage system - Construct laundry infrastructure with storage and drying compartments		
Hygiene	- Repair nonfunctioning sinks and increase the number of handwashing stations		
Waste Management	- Construct a new burn pit with shelter - Construct a new ash pit - Construction of new septic tank with leach field - Rehabilitate existing mortuary		
St. Timothy Hospital			
Water	- Rehabilitate the spring box - Construction of ground water storage tank - Construction of water tower with storage capacity of 6000 gallons - Construction of water transmission system from water source to storage tank and from storage tank to water tower - Construction of water transmission system from borehole to storage tank - Generator (50 kilovolts) - Solar-powered submersible pumps	$203,263.68	$148,527.00
Sanitation	- Construction of septic tank with leach field - Rehabilitation of sewer lines - Construction of toilets - Construction of laundry facility - Rehabilitation of storm water drainage system		
Hygiene	- Construction of water distribution system from water tower into hospital		
Waste Management	- Construction of new burn pit - Construction of placenta pit - Construction of an ash pit - Construction of fence for waste management section - Construction of mortuary		

* Includes major interventions only.

**Table 3:** “Basic” level of WASH services * in Liberian Government Hospital Bomi (LGH Bomi) and St. Timothy Hospital, pre- and post-intervention—Liberia, 2016 and 2018.

Domain	Basic Service	Pre-Intervention		Post-Intervention	
		LGH Bomi	St. Timothy	LGH Bomi	St. Timothy
Water	Water is available from an improved source on the premises	√	X	√	√
Sanitation	Improved sanitation facilities are usable, with at least one toilet dedicated for staff, at least one sex-separated toilet with menstrual hygiene facilities, and at least one toilet accessible for people with limited mobility.	X	X	√	√
Hygiene	Functional hand hygiene facilities (with water and soap and/or alcohol-based hand rub) is available at points of care and within five meters of toilets	X	X	√	√
Waste Management	Waste is safely segregated into at least three bins, and sharps are treated and disposed of safely	X	X	√	√
Environmental Cleaning	Basic protocols for cleaning are available, and staff with cleaning responsibilities have all received training	X	X	√	√

* Per Liberian WASH and Environmental Health Package in Health Facilities and SDG 6 standards, √ = available, X = not available.

**Table 4:** Costs of each component of renovations at both LGH Bomi and St. Timothy.

Component	LGH Bomi		St. Timothy	
	Absolute Cost (USD)	Percent of Facility Cost (%)	Absolute Cost (USD)	Percent of Facility Cost (%)
Renovation contracts	$174,155.78	50.0%	$194,057.00	50.8%
Equipment purchases	$55,250.00	15.8%	$67,989.00	17.8%
Service contracts for equipment (5 years)	$8,400	2.4%	$9,000	2.4%
Salary of WASH staff	$65,752.46	18.8%	$65,752.46	17.2%
Travel to the hospital sites *	$45,229.50	13.0%	$45,229.50	11.8%
Total costs	$348,796.74		$382,027.96	

* Travel includes transportation and daily sustenance allowance for technical staff supervising construction.

## Data Availability

Data available on request due to restrictions. The data presented in this study are available on request from the corresponding author. The data are not publicly available due to privacy.
